# Navigating the Complexity of Lung Cancer Surveillance Practices: Qualitative Pilot Study on Provider Perspectives

**DOI:** 10.2196/80659

**Published:** 2026-02-05

**Authors:** Jenny M Woo, Sydney Conover, Caroline Gray, Shipra Arya, Julie T Wu

**Affiliations:** 1 Division of Vascular Surgery, Stanford School of Medicine, 780 Welch Rd CJ350, Palo Alto, CA, 94304, United States, 1 408-759-0291; 2 Department of Oncology, Stanford School of Medicine, Palo Alto, CA, United States; 3 Center for Innovation to Implementation, VA Palo Alto Health Care System, Menlo Park, CA, United States; 4 Surgical Services, VA Palo Alto Health Care System, Palo Alto, CA, United States; 5 Stanford- Surgery Policy Improvement Research and Education (S-SPIRE) Center, Palo Alto, CA, United States

**Keywords:** surveillance, lung cancer, risk stratification, second primary lung cancer, patient-provider relationship, clinical decision-making

## Abstract

**Background:**

Surveillance is noted to be an important part of survivorship to detect recurrence and/or second primary lung cancer (SPLC) at a curable stage. However, current surveillance guidelines remain controversial, and the factors providers consider in clinical decision-making are neither well-defined nor consistently applied.

**Objective:**

In order to inform the qualitative protocol for a larger national study, this pilot study aimed to understand the factors that influence lung cancer surveillance and how providers view risk stratification as a potential tool to inform surveillance practices.

**Methods:**

Semistructured interviews were conducted between October 2023 and July 2024 with purposively sampled providers involved in treating and surveilling patients with lung cancer from the US-based Palo Alto Veterans Affairs Medical Center and Stanford Medicine and its affiliate clinics. Providers were recruited through both email outreach and in-person invitations. Interviews were transcribed by an external transcription service and analyzed through a qualitative inductive content analysis approach to identify themes.

**Results:**

In total, 11 physicians and 2 advanced practice providers (N=13) participated in interviews. The majority were from medical specialties (n=8, 61.5%), and the average number of years of practice as a provider was 9 years. A total of 3 themes were identified that describe the clinicians’ sentiments about current surveillance practices and how a risk stratification tool could be used in screening for recurrence and/or SPLC. Clinicians consider a variety of clinical and nonclinical factors (category 1: factors that influence clinical decision making) and highlighted limits of a risk stratification tool, including concerns about generalizability, accuracy, and validity (category 2: sentiments toward a hypothetical risk stratification tool). Finally, concerns were raised about how delivering risk stratification data might impact patient anxiety, misinterpretation, and adherence to surveillance plans (category 3: delivery of risk stratification data to patients).

**Conclusions:**

This qualitative analysis highlights the complexity of lung cancer surveillance decision-making and provider concerns about tool accuracy and delivery. While risk stratification tools may support surveillance decisions, their further development must address data quality, accuracy across diverse clinical and nonclinical risk factors, and effective patient-level data delivery. Doing so will facilitate the practical implementation of risk stratification tools to improve surveillance of SPLC and recurrence.

## Introduction

Surveillance is widely accepted to be an important part of lung cancer survivorship to help early detection of local recurrence and second primary lung cancer (SPLC) at a more curable stage. Guidelines by the American Society of Clinical Oncology, the National Comprehensive Cancer Network, and the European Society of Medical Oncology recommend surveillance imaging every 6 months for the first 2 years and then annually after those 2 years [1-3]. Despite these universal guidelines around surveillance, not all survivors receive surveillance imaging. One study found that two-thirds of 1288 patients did not receive recommended surveillance imaging [4], while another study reported that less than 1 in 5 patients out of 1537 at Veterans Health Administration received recommended surveillance imaging [5].

Current variability in imaging receipt may be due to a complex mix of factors, including uncertainty about the optimal frequency of surveillance, duration of surveillance, which findings warrant continued surveillance, or who would be a good candidate for surveillance, considering other comorbidities. Although surveillance imaging is a widely accepted and guideline-recommended practice, there is limited clinical trial evidence that increased surveillance improved survival, especially for patients with advanced non-small cell lung cancer (NSCLC) [6]. Moreover, patients’ comorbidities and overall life expectancy compel providers to balance the potential benefits of surveillance against its harms, including complications from biopsies of false positives and reduced quality of life from anxiety around scans [7,8]. How clinicians navigate these competing considerations remains unclear and may vary inconsistently across patients.

Tailored approaches to surveillance may benefit low-risk patients by reducing the frequency of surveillance, while focusing surveillance efforts on high-risk patients [6]. Risk calculators, developed using factors considered in retrospective evidence, can guide clinical judgment when current guidelines lack clear recommendations. For example, risk stratification based on malignancy risk is already used in primary lung cancer screening to identify high-risk patients and improve adherence to screening [9]. Applying a similar strategy to surveillance decision-making in survivors of lung cancer could help reduce variability and promote more consistent clinical practices.

Our study objective was to explore clinicians’ clinical decision-making practices around surveillance and to assess the acceptability of risk stratification tools to guide surveillance.

## Methods

### Study Design

This pilot qualitative interview study was designed to gather insights and perspectives from providers who treat lung cancer. The aim of this study was to identify the array of factors that providers consider for risk of recurrence and/or SPLC and to explore provider perceptions of a hypothetical risk stratification tool that would evaluate the risk of recurrence and/or SPLC.

To contextualize clinician feedback, the hypothetical risk stratification tool referenced in interviews was modeled after the validated Second Primary Lung Cancer–Risk Assessment Tool (SPLC-RAT) developed by Stanford Medicine [10]. SPLC-RAT generates a 5-year SPLC risk estimate using regression-based modeling of clinical and smoking history inputs (eg, age at diagnosis, cancer stage, histology, surgical resection, smoking status, pack-years, and time since cessation). In practice, such a tool could integrate with the electronic health record to automatically pull structured data (eg, age, gender, and race) and combine it with manually entered variables, similar to the integration of analogous tools for primary lung cancer screening [11]. While modeled after SPLC-RAT, the hypothetical risk stratification tool was unnamed to study participants to collect their broader perceptions of risk stratification tools.

### Recruitment

Providers were purposively identified and recruited from the Palo Alto Veterans Affairs (VA) medical center and Stanford Medicine and its affiliate clinics, all located within the United States. Eligibility criteria included licensed providers who are involved in treating and surveilling patients with lung cancer at these 2 health care systems. Eligible clinicians were invited to participate in the study through both email and in-person outreach. Initially, 1 provider from a thoracic oncology department was contacted, who then introduced the researcher (JMW) to other oncology providers in that department who were invited to be interviewed in person. The remaining providers were introduced by the study principal investigator (PI) (a thoracic oncologist; JTW) and were invited to participate via email.

### Interview Process

The interview guide was modeled on a previous study on machine learning prognostic algorithms and adapted to fit the research questions for this study [12]. The interview guide contained 9 questions surrounding provider surveillance practices in evaluating risk of recurrence and perspectives on a hypothetical risk stratification tool (Textbox 1). Mock interviews were conducted among the research team (JMW, JTW, and SC), and the interview guide was subsequently adjusted for clarity. Research questions asked to providers aimed to explore their clinical decision-making practices around lung cancer surveillance and to assess their perspectives on a hypothetical risk stratification tool to guide surveillance.

Textbox 1. **Interview questions.**

1. When considering stopping surveillance for a lung cancer survivor, some clinicians stop once a certain timeframe has passed (ie, five y), while others may prefer to continue surveillance indefinitely. When do you think clinicians should consider stopping surveillance indefinitely? When do you think clinicians should consider stopping surveillance imaging for a survivor?

- If they do not mention, ask: What are the reasons behind your opinion?
- Follow up: What events or circumstances prompt you to order a surveillance scan?
- If prompted for clarification or if the subject only discusses the time of diagnosis, mention that some clinicians are prompted by certain events or circumstances, such as multiple negative surveillance scans or declining functional status due to non-cancer-related causes.

2. How do you define risk of recurrence? Numerical risk? Or context of relative risk?

- Do you think about risk in concrete terms, as in quantitative numbers or relative terms (ie, this pt is higher risk than someone else)?

3. What factors do you use in assessing the risk of recurrence and/or second malignancy in the context of the patient’s overall health?

- I understand guidelines say to screen regularly, but in practice, there are other co-morbidities that can change your decision. Does this come to mind apart from the guidelines regarding quantitative risk assessment?
  - Do you use clinical intuition to modify guidelines?

4. What information about risk of recurrence and/or second malignancy do you usually share with a patient? [Do you have a discussion with a lung cancer survivor about whether or not to continue surveillance imaging?]

- If they do not describe this, ask: Some clinicians frame surveillance in terms of general risk, such as the high risk of recurrence for lung cancer. Others specifically tailor it to patient-specific factors, such as ongoing smoking and advanced stage at diagnosis. Where along that spectrum do you fall?

5. We are developing a computer program that can use a patient’s medical record data to estimate the patient’s risk of developing a second primary lung cancer. If you had access to a score that accurately estimated a 5-year risk of each of your patients -- for example, a 0.1% risk of cancer or a 6% risk of cancer -- how would you use this information in the care of the patient, if at all?

- If prompted for clarification of specific scenarios, specify: For example, a 60y patient diagnosed with early stage resected lung adenocarcinoma with a history of prior cancer before lung cancer and a 20 pk-yr smoking history has ~6% 5-year risk of SPLC. If that same patient did not receive resection and does not have a prior history of cancer, the risk reduces to 1.3%.
- If they do not mention, ask: Do you think it would prompt a discussion about surveillance practices?

6. Who on the care team should be the primary recipient of this information and how should it be incorporated into the clinical workflow? [Are you comfortable with the primary recipient of this information being a designated screening coordinator?]

- If prompted for clarification, mention that lung cancer screening has dedicated coordinators who triage patients based on risk assessment of lung nodule findings and that surveillance follow-up could follow a similar model

7. No prediction model will ever be perfect. A model could have many false positives, which in this case would be patients who are erroneously flagged as having a high risk of cancer. Alternatively, a model could have many false negatives, which in this case would be patients who are high risk of cancer but not flagged by the model. With that in mind, would you prefer to have a model that has more false negatives or more false positives? Why?

- If prompted to define “high risk of cancer,” it is whatever the clinician would consider high risk.

8. What concerns, if any, do you have about using computer-generated cancer risk predictions in the clinical care of your patients?

9. Do you have any current or prior experience using computer-generated models or cancer risk prediction tools to inform clinical decision-making?

- If the person answers “Yes,” ask: How does your experience impact the way you view the utility of computer-generated cancer risk predictions in clinical care?

After the main interview questions, demographic information including their years practicing in their specialty, specialty, age, gender, and race were collected.

Semistructured interviews with providers were conducted on Microsoft Teams by 2 trained female researchers (JMW and SC) and the female study PI (JTW). At each interview, verbal consent was obtained before the audio recording. Some study participants knew the PI before interviews, given her role as a thoracic oncology clinician at the health care systems participants were based. Some participants also had previous exposure to a previously developed SPLC risk stratification tool (SPLC-RAT) from Stanford Medicine, including as coauthors on the model development publication, which may have informed their responses to the hypothetical tool described in the interview guide. Interviews were conducted between October 2023 and July 2024, and audio files were transcribed by an external transcription service. The interviewers reviewed all transcriptions to verify accuracy. Participants were not contacted following the initial interview.

Our research team consists of oncology and surgical clinicians, along with health services researchers, including experts in qualitative methods and oncology. We value a comprehensive understanding of current health system landscapes and the importance of engaging with providers directly involved in patient care before developing future interventions and tools to enhance the quality of health services.

### Data Collection and Analysis

The research team aimed to interview at least 12 providers to support preliminary pilot findings; this has similarly been found sufficient for reaching a general level of saturation, or repetition of basic themes or findings [13]. When creating our participant sample, we also considered the concept of information power, which is defined as the capacity of a study’s sample size to effectively capture the unique experiences and insights of participants, particularly when the sample is specifically aligned with the research objectives [14]. Since the eligibility criteria created a smaller, well-defined sample of eligible participants, a smaller sample size was sufficient to provide exploratory pilot data that addressed our research questions. Moreover, since interview questions were highly specific, with multiple follow-up probes, and there was depth to the answers, a wide array of insights was captured despite a smaller sample.

Interview transcripts were uploaded to Microsoft Excel. Data analysis was completed through an inductive content analysis approach. First, familiarization with the data was conducted by 2 researchers (JMW and JTW), after which a list of initial codes was identified. During this process, the researchers met weekly to discuss and determine consensus, creating the initial codebook, which contained preliminary codes and definitions, and to adapt the codebook as needed. The finalized codebook was further reviewed externally by the qualitative methods methodologist (CG), providing expert validation and addressing potential biases that may have arisen from the internal coding team. Once the codebook was agreed upon, the rest of the interviews were coded (JMW and SC). Finally, codes were analyzed and organized into groups to identify overarching themes. Themes were defined, and supporting data that were already analyzed were summarized into these larger themes.

### Ethical Considerations

The study was approved by the Stanford University Institutional Review Board (IRB #67922) as an expedited review with a waiver of documentation of informed consent. VA Local Union Notification was obtained for VA employees. Oral information about the study was given before verbal informed consent. This included details about the study’s purpose, risk, and benefits of participation, confidentiality protections, and participants’ rights to withdraw at any time. All participants provided verbal consent for participation before audio recording. All data were deidentified before transcription using a coding system. Only the interviewers (JMW, SC, and JTW) had access to the code key. Transcriptions and audio recordings were stored in a secure and access-restricted folder on a server behind the VA firewall, accessible only to the research team. Participants did not receive any compensation for their participation.

## Results

### Participants

Of the 29 eligible providers recruited to participate, 11 physicians and 2 advanced practice providers (N=13) who were specifically involved in treating and surveilling patients with lung cancer were interviewed. The interviews ranged from 14 to 30 minutes. The average number of years the participants have been practitioners was 9 (SD 5.52, range 3‐20) years. The majority of participants were medical specialists (n=8, 61.5%), while the remaining 38.4% (n=5) were interventional specialists. Additional demographic information is provided in Table 1.

**Table 1.** Demographic characteristics of providers (N=13).

Characteristics	Value
Age (y), mean (SD; range)	42.5 (5.75; 34-50)
Sex, n (%)	
Female	9 (69.2)
Male	4 (30.8)
Race, n (%)	
White	6 (46.1)
African American	1 (7.8)
Asian	6 (46.1)
Ethnicity, n (%)	
Hispanic, Latino/a/x, or Spanish Origin	0 (0)
Specialty, n (%)	
Interventional	5 (38.4)
Medical	8 (61.5)
Average time as a practitioner (y), mean (SD; range)	9 (5.52; 3-20)

### Themes

Findings clustered around three main themes: (1) factors that influence clinical decision making, (2) sentiments toward a hypothetical risk stratification tool, and (3) delivery of risk stratification data to patients. Themes and illustrative quotes are displayed in Table 2.

**Table 2.** Themes and representative quotes.

Overarching theme and subcategory	Definition	Representative quotes
Factors that influence clinical decision-making (for surveillance)		
Clinical risk factors	Clinical risk factors providers consider for surveillance, including cancer-specific and non–cancer-specific factors.	“I would probably consider smoking history in addition to the patient’s medical history. Maybe other medical illnesses we need to consider, different types of cancers and pathologies” [Interventional Specialty, practicing for 10 y] “What the patient’s overall life expectancy is in respective to the diagnosis” [Medical Specialty, practicing for 1.5 y] “With older people over the age of 75, they have competing risks. Not just their lung cancer recurrence but also dying from other causes so we certainly take that in account and a patient’s ability to continue coming in. It’s really a shared decision making with the patient” [Medical Specialty, practicing for 12 y]
Nonclinical risk factors	Nonclinical risk factors that providers consider such as patients’ social circumstances and sentiments toward surveillance. Some social circumstances include differing emotional capacities, financial situations, and insurance coverage.	”[There’s] still a lot of anxiety result[ing] from living as a cancer survivor” [Medical Specialty, practicing for 21 y]
Sentiments toward a hypothetical risk stratification tool		
Positive sentiments	Providers’ positive sentiments toward a hypothetical risk stratification tool	“I think these risk calculators could be very helpful because it gives us concrete idea of the risk level and if it tells us of a very low risk level, it can give us some confidence in doing less surveillance imaging” [Medical Specialty, practicing for 8 y]
Provider concerns	Concerns providers have toward a hypothetical risk stratification tool. This includes concerns about the tool’s generalizability, accuracy, and validity.	“I’d have to trust the model and have to trust the data that is going into it to make sure I would be convinced that the information would be helpful to me” [Medical Specialty, practicing for 15 y] “I think a computer-generated model will certainly have more pitfalls in poor and less fortified areas. You’re going to have a lot of data for the rich folks and not so much data on the poor folks. This might be a serious social justice element to that – who gets screened for cancer, people who can afford a house in Palo Alto? If you live in Nevada, maybe not so.” [Interventional Specialty, practicing for 10 y]
Views on false positives versus false negatives	Provider views on which error (type I or type II) they would choose between in a model setting.	“The repercussions of missed opportunity to diagnose recurrence earlier is worse than the risk of overtreating” [Interventional Specialty, practicing for 14 y] “Could do some Markov modeling to see ideal threshold. There could be downsides to over biopsying benign things. I would err on the side of flagging people” [Medical Specialty, practicing for 4 y]
Provider concerns about delivery of risk stratification data to patients	Providers’ concerns about how patients would receive the risk stratification data.	“Making sure that they understand the importance of coming back for their scans regularly” [Interventional Specialty, practicing for 5 y] “I think it would be valuable to speak to that patient about the risk of radiation, risk of recurrence of cancer, and really put the numbers in front of them” [Interventional Specialty, practicing for 10 y]

### Factors That Influence Clinical Decision Making

**Figure 1.**
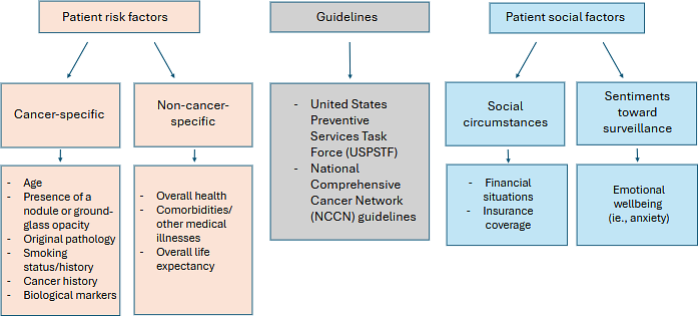
Factors that influence provider decisions on surveillance. This figure describes a summary of the factors mentioned that influence provider decisions on lung cancer surveillance. The first row of headings is different overarching groups of factors. Following each heading (“patient risk factors,” “guidelines,” and “patient social factors”) are arrows pointing to further specific categories under the respective headings. For the “patient risk factors” and “patient social factors” sections, there is a second set of arrows that are pointing to specific examples of factors that were noted by the participants.

There are a multitude of factors that providers consider that can influence their clinical decision making, falling into 2 categories, clinical factors (patient risk factors and guidelines) and nonclinical factors (patients’ social factors; Figure 1).

#### Clinical Factors (Patient Risk Factors and Guidelines)

When providers were asked about what factors they consider for surveillance, they first mentioned cancer-specific factors (patient’s age, presence of a nodule or ground-glass opacity, original pathology, smoking status and history, and medical history). They also explained that they consider cancer history (stage of cancer, history of lung cancer, family history of cancer, and time from treatment) as well as biological markers (presence of mutations, molecular markers, and lymph node status) when considering surveillance for a specific patient.

Besides these factors, they also described how they consider guidelines and published data. Many providers mention a 5-year cutoff for surveillance unless there is a presence of a nodule, while others stated they screen every year indefinitely. The United States Preventive Services Task Force and National Comprehensive Cancer Network guidelines were mentioned as resources they use as well. When asked if they currently use a risk stratification tool for risk of recurrence and/or SPLC, the providers denied using one.

Beyond the guidelines and published data, providers listed other non–cancer-specific clinical factors, such as the patient’s overall health, comorbidities, other medical illnesses, and overall life expectancy. One provider expressed how:

*...with older people over the age of 75, they have competing risks. Not just their lung cancer recurrence but also dying from other causes so we certainly take that in account and a patient’s ability to continue coming in. It’s really a shared decision making with the patient.*

[P04]

One provider mentioned how they look at life expectancy tables to help decide if focusing on lung cancer surveillance is in the patient’s best interest.

#### Nonclinical Factors (Socioeconomic and Patient Sentiments)

Factors such as patient social circumstances and patient sentiments toward surveillance were highlighted. One provider stated,

*People have a certain bandwidth for their healthcare, and I think they should be able to put their energy on what specific condition they should focus on [ie, heart condition or diabetes].*

[P01]

They explained that patients’ factors, such as differing emotional capacities, financial situations, and insurance coverage, all play a role in their clinical judgment on whether surveillance is beneficial. For instance, it was mentioned,

*I also tell them things like I don’t know if their insurance will cover surveillance after five years and things like that and then generally if they want to, then I’ll order the scan.*

[P08]

The provider explains that financial capability is also a part of the shared decision-making process in considering surveillance. Another provider described how they consider “healthcare spending [and] patients’ bank accounts” (P10) as well.

Emotional well-being, particularly patient anxiety, was stated a few times. One provider explained how there’s “still a lot of anxiety result[ing] from living as a cancer survivor” (P07). They noted there are significant emotional challenges that patients face as survivors of lung cancer and expressed great caution with how they bring up the topic of risk of recurrence and/or SPLC with their patients. One provider shared how “...many of them have anxieties, of course, some of them won’t want the scans sooner and then some people don’t want to know” (P11).

Another provider brought up a similar sentiment, stating:

*There is a lot of anxiety about just having nodules knowing that one of them turned into a lung cancer group [in the past].*

[P08]

The providers explained how this is a shared decision-making process with their patients, acknowledging patients’ socioeconomic and emotional bandwidths. One provider acknowledged that at times, they’ll need to follow how the patient feels and stated:

*If the patient decides that they don’t want to do surveillance anymore then it is a discussion and then we will stop.*

[P11]

### Sentiments Toward a Hypothetical Risk Stratification Tool

**Figure 2.**
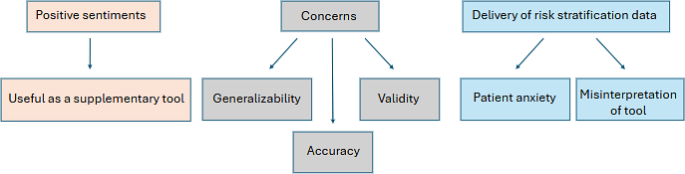
Provider sentiments on a risk stratification tool. This figure describes a summary of the provider sentiments on a hypothetical risk stratification tool. The first row of headings is different overarching groups of sentiments. Following each heading (“positive sentiments,” “concerns,” and “delivery of risk stratification data”) are arrows pointing to further explanations and examples of sentiments that were noted by the participants. For the “delivery of risk stratification data,” the arrows are pointing to outcomes providers expressed concerns of in relation to how the risk stratification data is delivered to patients.

When asked about a hypothetical risk stratification tool that can use a patient’s medical record to estimate a 5-year risk of recurrence and/or SPLC, providers were generally positive, but did express concerns surrounding a risk stratification tool (Figure 2).

#### Positive Sentiments

Many providers expressed interest in the hypothetical risk stratification tool, especially its role in surveillance practices past the 5-year mark, which they consider the cutoff for surveillance in survivors of lung cancer without a nodule as it would be “useful for determining whether [they] should keep surveying after 5 years” (P08). Having a risk stratification tool could be beneficial for providers,

*...because it gives [a] concrete idea of the risk level and if it tells of a very low risk level, it can give some confidence in doing less surveillance imaging.*

[P10]

While these providers expressed positive attitudes toward this hypothetical risk stratification tool, the majority stated that although it could be useful as a supplementary tool to clinical decision making, they would not rely solely on it due to various concerns outlined below.

#### Provider Concerns

Many providers expressed concerns about the generalizability, accuracy, and validity of the tool. They questioned the data the model would be using, with 1 provider suggesting:

*...[they would] have to trust the data that is going into it to make sure [they] could be convinced that the information would be helpful.*

[P13]

They expressed hesitancies if a tool could consider all the factors they consider and how representative of the patient population the data would be.

Regarding generalizability, 1 provider mentioned the potential shortcoming in the data surrounding socioeconomic status, stating:

*...[the] model will certainly have more pitfalls in poor and less fortified areas. You’re going to have a lot of data for the rich folks and not so much data for poor folks. That might be a serious social justice element to that – who gets screened for cancer, people who can afford a house in Palo Alto? If you live in Nevada, maybe not so.*

[P02]

Not only that, underrepresentation of racial and gender disparities was also highlighted as a concern, with 1 provider expressing that,

*...[they] worry a bit about racial or gender disparities in terms of information that models are given and whether that will actually apply to all patients.*

[P08]

Due to the concerns that the data may not be representative and comprehensive enough, the providers suggested that the model may not be generalizable to their patients and thus may not be a tool they can fully rely on in their clinical decision-making.

Concerns with the accuracy and validity of the input data for the tool were also shared. A participant explained,

*...as you do this [with] large amounts of data, things that might not be apparent otherwise can come forward. That’s where we have the challenge of, is it really there or is it not really there, but you know you’re not going to have most people though there will be some who ask about all those little details.*

[P07]

Other concerns providers highlighted were the limitations of relying on data from electronic medical records. A provider stated that “there is lots of variability in the patient data like the dates of diagnoses” (P09) and how “the date of diagnosis is not discrete and consistent” (P09). One provider illustrated that,

*...many patients have incomplete follow-up and survival data which could bias your observed outcomes and could result in an inaccurate model.*

[P10]

Therefore, instead of being a primary tool to depend on, the providers emphasized that this tool should have a “disclaimer that of course [is] not predictive” (P12) of whether a patient has a recurrence and/or SPLC and should be used as a supplementary check.

#### Views on False Positives Versus False Negatives

Providers also discussed their perspectives on false positives and false negatives in a tool, with most providers preferring a model threshold that errs on the side of more false positives. One provider explained,

*The repercussions of missed opportunity to diagnose recurrence earlier is worse than the risk of overtreating.*

[P03]

Other providers, while still agreeing that false positives were better than false negatives, shared that there should be “some Markov modeling to see [an] ideal threshold” (P06) to help minimize the difference. Many providers illustrated that this approach of more false positives would be useful in flagging patients and then verifying if the finding is accurate, but false positives would falsely worry patients who did not have lung cancer, and that “there could be downsides to over biopsying benign things” (P06).

### Delivery of Risk Stratification Data to Patients

When asked whether patients should directly receive the risk stratification tool data, most of the providers emphasized that patients should not directly receive it due to the potential for misinterpreting the numbers and developing anxiety about their health status. Many of the providers explained that they generally do not give quantitative values involving risk prediction as they want to avoid worrying their patients. They also worry that if they gave specific numbers, it would not truly resonate with the patient, as they would not understand the numbers in depth. One provider puts it as sharing enough in “making sure that they understand the importance of coming back for their scans regularly” (P08). There were a few providers who stated that they would share exact numbers to patients if the patients pushed for those numbers or used the numbers “...to speak to that patient about the risk of radiation, risk of recurrence of cancer, and really put the numbers in front of them” (P02).

## Discussion

### Principal Findings

This qualitative pilot study interviewed specialists from 2 medical systems about their current practices surrounding surveillance for risk of recurrence and/or SPLC, their attitudes toward a hypothetical risk stratification tool, and how they would deliver risk stratification data to their patients. Surveillance decisions were found to incorporate diverse clinical and nonclinical factors beyond current guidelines, which may explain variability in imaging receipt observed in population-based studies [15]. While participants showed interest in risk stratification tools to systematize decision-making, they raised concerns about the tool’s generalizability, accuracy, validity, and content delivery. These concerns highlight key areas for future tool and intervention development. These pilot findings will also inform the development of a future national survey study of physician attitudes and perspectives of an adapted risk stratification tool for SPLC and lung cancer surveillance.

Providers emphasized how their surveillance plans go beyond following current guidelines, incorporating both clinical and nonclinical risk factors. They consider patients’ overall well-being, comorbidities, and sentiments toward surveillance, tailoring efforts to reduce adverse outcomes, such as complications or reduced quality of life [7,8]. Most participants highlighted the importance of shared decision-making, which has been shown to improve knowledge surrounding lung cancer screening and be positively associated with intentions to go through screening again [16]. This personalized approach allows providers to create comprehensive patient-centered surveillance plans.

With innovations, such as computer models and artificial intelligence (AI) in cancer care, understanding provider acceptance and opinions toward a computer-generated risk stratification tool is important [17]. This feedback provides risk stratification tool developers with the necessary elements and concerns clinicians are likely to have. Given the complexity of factors in lung cancer recurrence and SPLC, such models could guide and standardize surveillance by identifying high-risk patients while reducing unnecessary low-dose computed tomography scans for low-risk individuals.

Providers expressed interest in a hypothetical risk stratification tool for improving clinical care, but stated it was not robust enough to be used independently and emphasized they trust their clinical intuition above all. They viewed it as a supplemental tool that could provide additional validation to their clinical decision-making, citing limitations such as the data quality and model accuracy. Providers emphasized the importance of inclusive input data, particularly for underrepresented groups based on income, race, and gender. This has been shown to be a significant barrier to the widespread adoption of AI models in cancer care [18]. Accuracy concerns were also raised, such as overestimating risk in low-risk individuals or failing to identify high-risk patients. Given these limitations, most providers viewed such tools as secondary to clinical intuition. They stressed that the tool’s output should not drive decisions independently, and that providers should understand the input factors and rely on their expertise to override the tool if inaccuracies arise.

Delivery of risk information was another key concern. Most providers indicated that they would avoid sharing exact risk stratification numbers with their patients, citing concerns about potential misinterpretation and anxiety. Studies have shown that patients with cancer already have scan-associated anxiety, and adding to this underlying anxiety could negatively influence their adherence to getting necessary surveillance scans [19,20]. Instead, providers have suggested that they could use the risk stratification tool to initiate conversations about risk without focusing on specific numbers. These sentiments from providers shed light on the importance of considering how patients will receive risk stratification tool information before sharing it, and in response, sharing enough information to ensure that the patient can continue to pursue the best care in a well-informed manner. Although providers viewed this tool as supplementary, they agreed that it could be a helpful double-check for their own clinical judgment and prompt alternative surveillance strategies, such as adjusting scan frequency. Ultimately, providers emphasized that with a tool or not, their goal for patients is to adhere to the most clinically accurate and effective plan to prevent recurrence and/or SPLC.

### Limitations

First, the small sample size limits the transferability of findings to lung cancer survivorship specialists nationwide. However, as a pilot study, it gathered perspectives across various settings, including academia, the VA, and satellite clinics, which may have broadened the findings beyond a single clinic or hospital. Nonetheless, these results will inform our planned national study, which will produce more transferable findings. Second, participants were from an affluent, research-focused region, and providers may be more receptive to technology and innovation than those at other locations. This initial pilot study was also limited by recruitment reach. Future work would benefit from the inclusion of a more diverse group of lung cancer providers from various geographic locations, institutions, and hospital settings across the United States, particularly those in rural areas where access to new technology and innovation may be limited or less emphasized. Third, some participants knew the PI before interviews, given her role as a thoracic oncologist, and some participants had previous exposure to SPLC risk stratification tools. Due to narrow eligibility criteria and the interconnectedness of these 2 health care systems, this was inevitable and had the potential to impact study participants’ responses. Future work will gather perspectives from a national sample of providers to broaden findings and make them more transferable. Fourth, providers’ perceptions were reported based on a hypothetical tool, rather than one they have used or trialed, limiting the applicability of findings. While the hypothetical risk stratification tool concept was explained to providers and some had previous knowledge of risk stratification tools, participants were still inferring what the tool could or could not do. Future work should provide a detailed explanation of a developed risk stratification tool, outlining its exact functionality within surveillance.

### Conclusions

This study illustrates the complexity of lung cancer surveillance decision-making, which may contribute to variability in surveillance imaging among survivors. While risk stratification offers a systematic approach to guide decisions, uptake is limited by concerns about data quality, tool accuracy, and delivery and applicability to individual patients. Addressing these issues can inform the development and implementation of more acceptable and effective risk stratification tools.

## Abbreviations:

AI: artificial intelligence
SPLC: second primary lung cancer
SPLC-RAT: Second Primary Lung Cancer–Risk Assessment Tool
VA: Veterans Affairs
